# Development of a novel predictive model for lymph node metastasis in patients with endometrial endometrioid carcinoma

**DOI:** 10.1186/s12885-022-10437-2

**Published:** 2022-12-20

**Authors:** Xingdan Guo, Chunhua Lin, Jing Zhao, Mi Tang

**Affiliations:** 1 Department of Pathology, Hunan Provincial Maternal and Child Health Care Hospital, Changsha, China; 2 Department of Gynecological Oncology and Cervical Lesion, Hunan Provincial Maternal and Child Health Care Hospital, Changsha, China

**Keywords:** Histopathologic predictive model, Lymph node metastasis, Survival, Endometrioid carcinoma

## Abstract

**Background:**

Globally, the burden of endometrial endometrioid carcinoma (EEC) increases annually. However, the histological grade of EEC remains unelucidated. We developed a novel model for predicting lymph node metastasis (LNM) in patients with endometrioid carcinoma (EC), which has not been well established.

**Methods:**

A total of 344 patients with EEC were classified into training (*n* = 226) and validation (*n* = 118) cohorts. To develop a nomogram to predict LNM, independent predictors were defined using univariate and multivariate regression analyses. The calibration curve, area under the decision curve analysis (DCA), and receiver operating characteristic curve were used to evaluate the performance of the nomogram.

**Results:**

Independent predictors of LNM in EC were identified in the univariate analysis, including mitosis; microcystic, elongated, and fragmented patterns; lymphovascular invasion (LVI); necrosis; and high-grade pattern. Mitosis, LVI, and high-grade pattern remained independent predictors of LNM in multivariate analysis. An LNM nomogram that was constructed by incorporating the five predictors showed reliable discrimination and calibration. DCA showed that the LNM nomogram scoring system had significant clinical application value. In addition, a high nomogram score (score > 150) was a significant prognosticator for survival in both LNM-positive and LNM-negative ECs.

**Conclusions:**

Our novel predictive model for LNM in patients with EC has the potential to assist surgeons in making optimal treatment decisions.

## Background

Endometrioid carcinoma (EC) is the most common histological subtype of endometrial carcinoma. The Silverberg and International Federation of Obstetrics and Gynecology (FIGO) grading systems are widely used in routine practice for grading endometrial endometrioid carcinoma (EEC). Although both grading systems have prognostic significance, they focus only on tumor construction and nuclear atypia, ignoring other histologic features such as tumor stroma type, invasion pattern, and tumor necrosis. In the 5^th^ edition of the World Health Organization classification of female genital tumors, according to the Silva system, female genital tumors are considered the most informative histologic factors for cervical human papillomavirus-associated adenocarcinoma, with a clear stratification between lymph node metastasis (LNM) and prognostic risk. However, the histological grading of endometrial carcinoma still follows the original Silverberg or FIGO system.

In addition to the features mentioned in the Silverberg and FIGO grading systems, several other histologic features have also been proposed [1, 2], with the most promising being a microcystic, elongated, and fragmented (MELF) invasive pattern. This pattern has been associated with LNM. Tumor necrosis has also been associated with invasiveness, which is in turn related to worse clinical outcomes in many malignancies [3]. Tumor stroma types, including the inflammation type (rich in infiltrating lymphocytes), fibroblastic type (desmoplastic reaction), or myxoid type (immature myxoid change), have been found to play important biological roles in cancer development [4]. Thus, these proposed factors have not yet been incorporated into a grading system, which limits clinicians’ ability to predict LNM in patients with EEC.

Thus, this study aimed to build a novel nomogram of histologic variables as risk factors for predicting LNM in patients with EEC to aid in the clinical decision-making process for such patients.

## Methods

### Patients

This retrospective study included 344 consecutive patients diagnosed pathologically with pT1 EEC who underwent radical surgery between January 2010 and December 2018 at the Hunan Provincial Maternal and Child Health Care Hospital in Changsha, China. The 344 included patients were allocated randomly to a training cohort (*n* = 226) and a validation cohort (*n* = 118) at a 2:1 ratio using a data splitting approach. Patients who underwent preoperative treatment or had dedifferentiated or undifferentiated EC were excluded.

### Assessment of clinicopathological factors

Information on age and LNM were obtained from the original reports. All the original hematoxylin–eosin-stained slides were reassessed histologically, and the following variables were considered: (i) lymphovascular invasion (LVI), defined as the presence of tumor cells within the endothelial-lined channels; (ii) necrosis, defined as the presence of necrotic granular and eosinophilic material, which may be accompanied by apoptosis, ghost cells, and neutrophils; and (iii) a high-grade pattern defined as solid, micropapillary, fused glands or single cells infiltrating the desmoplastic stroma. Silverberg grading was based on the architecture (majority glandular = 1, papillary = 2, solid = 3), nuclear atypia (mild = 1, moderate = 2, severe = 3), and mitotic activity in 10 high-power fields (0 to 9 = 1, 10 to 24 = 2, ≥ 25 = 3); the added score determined the grade (G1, 3–5; G2, 6–7; G3, 8–9). The FIGO grading was based on the percentage of solid components (G1: < 5%, G2: 5% to 50%, G3: > 50%); severe atypia warranted upgrading to the architectural FIGO grade (1 to 2 or 2 to 3).

### Statistical analyses

Univariate and multivariate logistic regression analyses were performed to identify the significant independent factors for predicting LNM. Variables with *P* < 0.1 in the univariate analysis were included in the multivariate analysis. *P*-values were obtained based on two-tailed statistical analyses, and the significance level was set at 5% (*P* < 0.05). R software (version 4.1.0, www.r-project.org) was used for all the statistical analyses. The R statistical packages “rms,” “barplot,” “Hmisc,” “MASS,” and “pROC” were used to plot the distributions of the risk scores and metastasis, draw the calibration and receiver operating characteristic curves, and build a nomogram, while the “rmda” package was used to draw the decision curve analysis (DCA) curves and the “forestplot” package to draw the forest plots and Kaplan–Meier curves.

## Results

### Demographic and clinicopathological findings

The baseline clinicopathological characteristics of the 344 included patients are summarized in Table 1. LNM was observed in 76 (20.6%) cases. The mean ± standard deviation for patient age was 63.4 ± 11.2 years. LNM showed a positive association with high-grade nuclei, MELF pattern, high-grade pattern, LVI, necrosis, and mitosis. The resection margins were negative for all tumors. Age differences, T stage and tumor size, were not statistically significant.

**Table 1:** Associations between clinicopathological features and LNM

Variable		All patients	LNM		*P* value
			Absent No.(%)	Present No.(%)	
Age (years)^*^		63.4 ± 11.2 [35—91]	62.2 ± 10.4 [35—89]	64.1 ± 10.5 [38—91]	0.875
T stage	T1	171	137 (80.1)	34 (19.9%)	0.734
	T2	197	155 (78.7)	42 (21.3)	
Tumor size (mm)		33.2 ± 7.2 [12.5—43.5]	32.2 ± 7.4 [13.5—38.5]	34.1 ± 7.5 [12.5—43.5]	0.123
High grade nuclear	Absent	281	241 (85.8)	40 (14.2)	0.014
	Present	63	46 (73.0)	17 (27.0)	
MELF	Absent	310	269 (86.8)	41 (13.2)	< 0.001
	Present	34	18 (52.9)	16 (47.1)	
Stroma reaction	Inflammatory	56	49 (87.5)	7 (12.5)	0.100
	Fibrosis	230	195 (84.8)	35 (15.2)	
	Myxoid	58	43 (74.1)	15 (25.9)	
Histology grade pattern		22.5 ± 19.4 [0.0—75.0]	19.3 ± 13.7 [0.0—70.0]	38.9 ± 17.4 [3.0—75.0]	< 0.001
Lymph-vascular invasion	Absent	220	200 (90.9)	20 (9.1)	< 0.001
	Present	124	87 (70.2)	37 (29.8)	
Necrosis	Absent	293	260 (88.7)	33 (11.3)	< 0.001
	Present	51	27 (52.9)	24 (47.1)	
Mitosis		7.3 ± 3.1 [1.0—16.0]	6.8 ± 2.9 [1.0—14.0]	9.8 ± 2.9 [3.0—16.0]	< 0.001
FIGO	G1	51	48 (94.1)	3 (5.9)	< 0.001
	G2	185	165 (89.2)	20 (10.8)	
	G3	108	74 (68.5)	34 (31.5)	
Silverberg	G1	86	82 (95.3)	4 (4.7)	< 0.001
	G2	175	149 (85.1)	26 (14.9)	
	G3	83	56 (67.5)	27 (32.5)	

*LNM* lymph node metastasis, *MELF* microcystic, elongated, and fragmented, *FIGO* International Federation of Obstetrics and Gynecology

^*^Data are presented as mean ± standard deviation

### Evaluation and validation of the LNM prediction nomogram

**Fig. 1:**
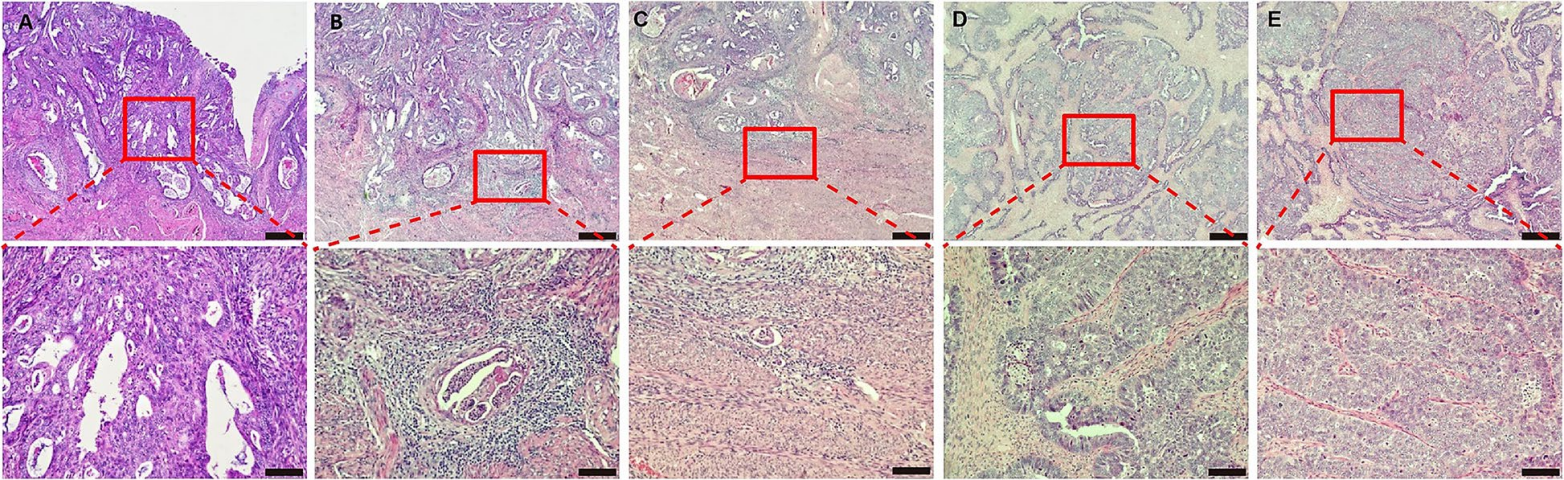
Histological features of endometrioid carcinomas. Representative photomicrographs of **A** mitosis; **B** a microcystic, elongated, and fragmented pattern; **C** lymphovascular invasion; **D** tumor necrosis; **E** and a high-grade pattern; SCALE BAR = 500μm in low power field, SCALE BAR = 100μm in high power field

**Fig. 2:**
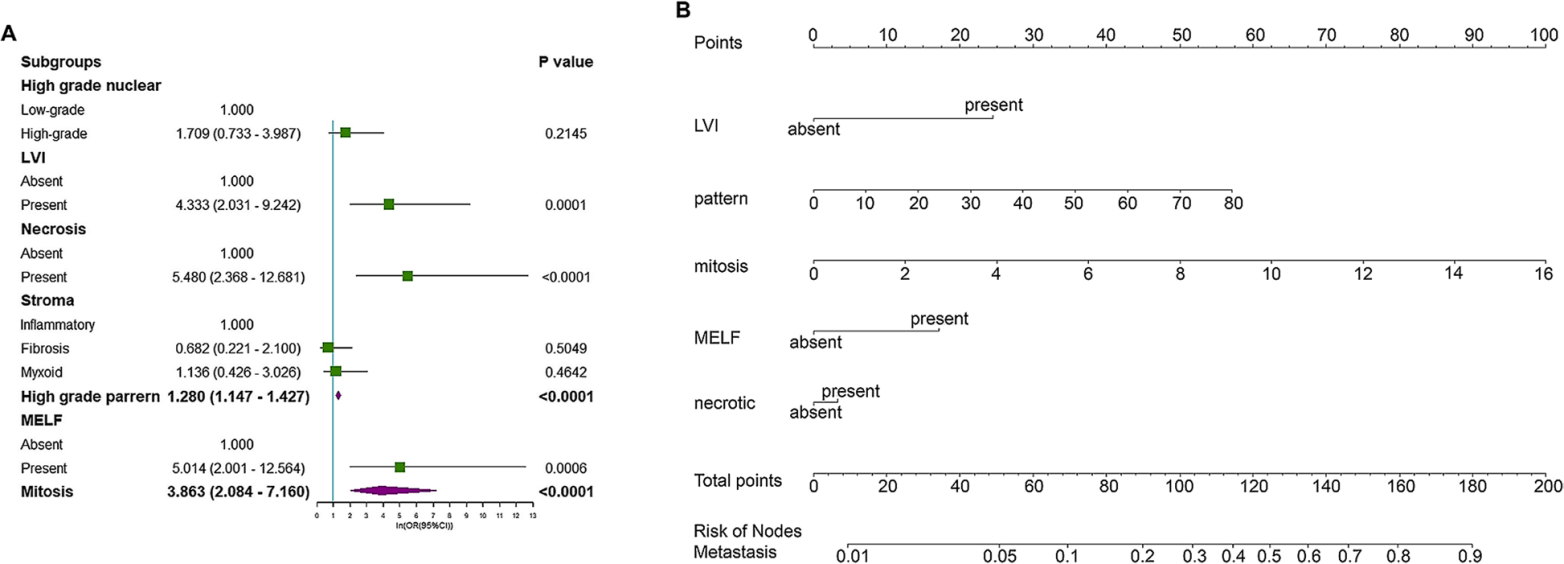
Predicted model of lymph node metastasis (LNM). **A** Forest plots to decipher the risk factors associated with LNM in the multivariate logistic regression analysis. **B** Newly developed nomogram for predicting LNM in patients with endometrial endometrioid carcinoma

The 344 included patients were allocated randomly to a training cohort (*n* = 226) and a validation cohort (*n* = 118) at a 2:1 ratio using a data splitting approach. According to the results of the univariate logistic regression analysis for the training cohort, five factors, including mitosis (Fig. 1A), MELF pattern (Fig. 1B), LVI (Fig. 1C), necrosis (Fig. 1D), and high-grade pattern (Fig. 1E), were linked to LNM status (Fig. 2A). Mitosis, LVI, and high-grade pattern remained independent predictors of LNM in the multivariate analysis (Table 2, Fig. 2B).

**Table 2:** Multivariate logistic regression analysis of lymph node metastasis

	Training cohort (*n* = 226)		Validation cohort (*n* = 118)	*P* value
	OR (95% CI)		OR (95% CI)	
Mitosis	3.202 (1.650–6.214)	0.0006	4.927 (2.353–7.856)	0.0013
MELF pattern				
Absent	1		1	
Present	1.977 (0.508–7.695)	0.056	1.689 (0.938–9.100)	0.325
LVI				
Absent	1		1	
Present	2.650 (1.121–6.262)	0.0263	4.912 (0.833–8.951)	0.078
Necrosis				
Absent	1		1	
Present	1.142 (0.307–4.244)	0.842	1.147 (0.186–7.052)	0.881
High-grade pattern	1.217 (1.065–1.389)	0.0037	1.795 (1.317–2.446)	< 0.001

*OR* odds ratio, *CI* confidence interval, *MELF* microcystic, elongated, and fragmented, *LVI* lymphovascular invasion

**Fig. 3:**
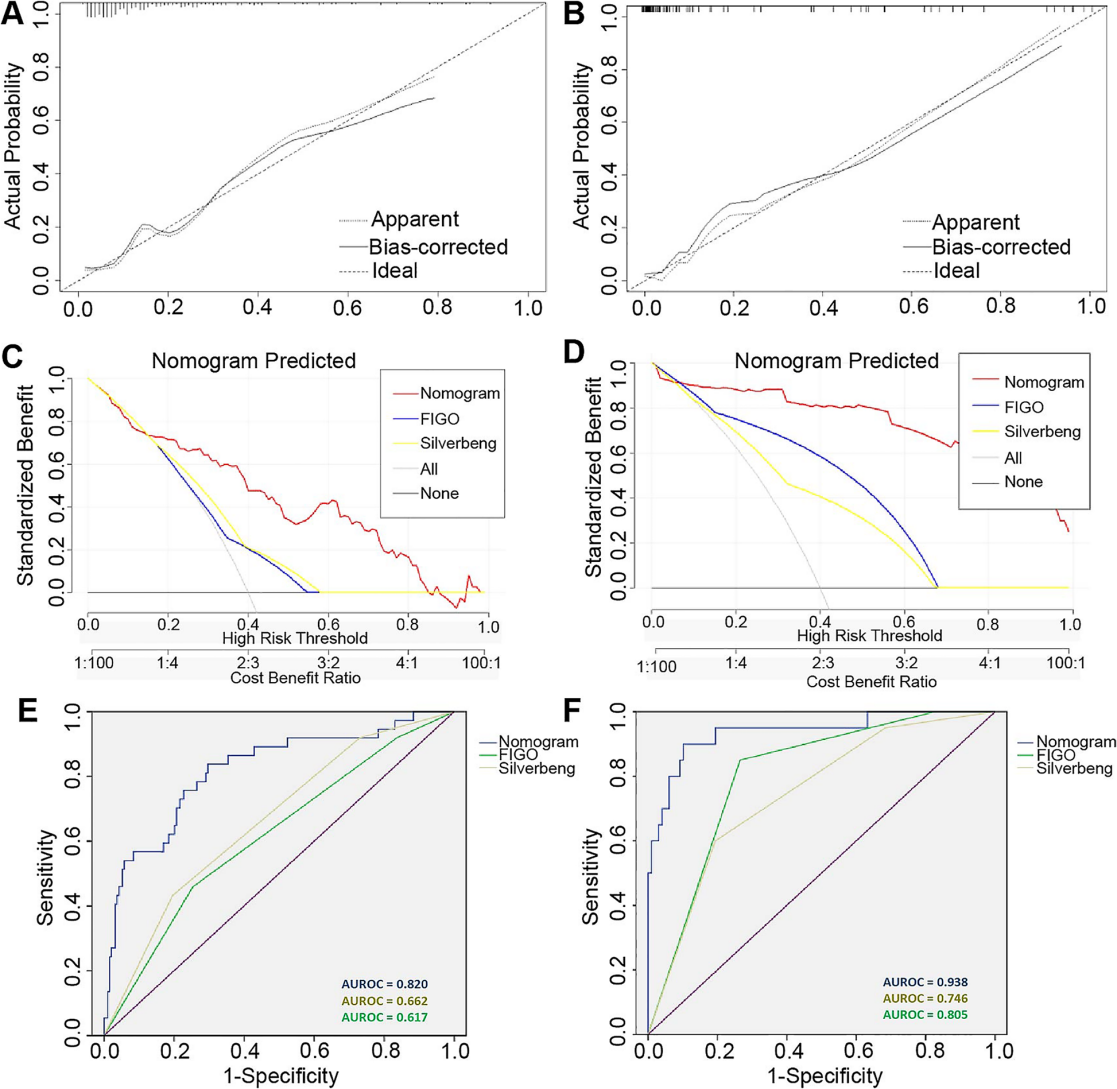
Calibration curve for predicting lymph node metastasis (LNM) in patients with endometrial endometrioid carcinoma (EEC) in the **A** training and **B** internal validation cohorts. Decision curve analysis of the nomogram and other systems for predicting LNM in patients with EEC in the **C** training and **D** internal validation cohorts. The gray line and black line represent the assumption regarding all patients with and without LNM, respectively. Comparison of predictive accuracy between the nomogram and other systems in the **E** training and **F** internal validation cohorts

Compared with the FIGO or Silverberg system, the calibration curve of the LNM nomogram was consistent with the standard curve, indicating greater reliability of the predictive ability of the nomogram (Fig. 3A, B). The C-indices of the nomogram were 0.820 (95% confidence interval [CI]: 0.740–0.900) and 0.938 (95% CI: 0.871–0.986) in the training and validation cohorts, respectively (Fig. 3C, D). The DCA curves for the LNM nomogram in the training and validation cohorts are shown in Fig. 3E and F, respectively. The DCA curves of the nomogram showed higher net benefits, indicating good clinical value.

### Survival impact of the prediction nomogram

**Fig. 4:**
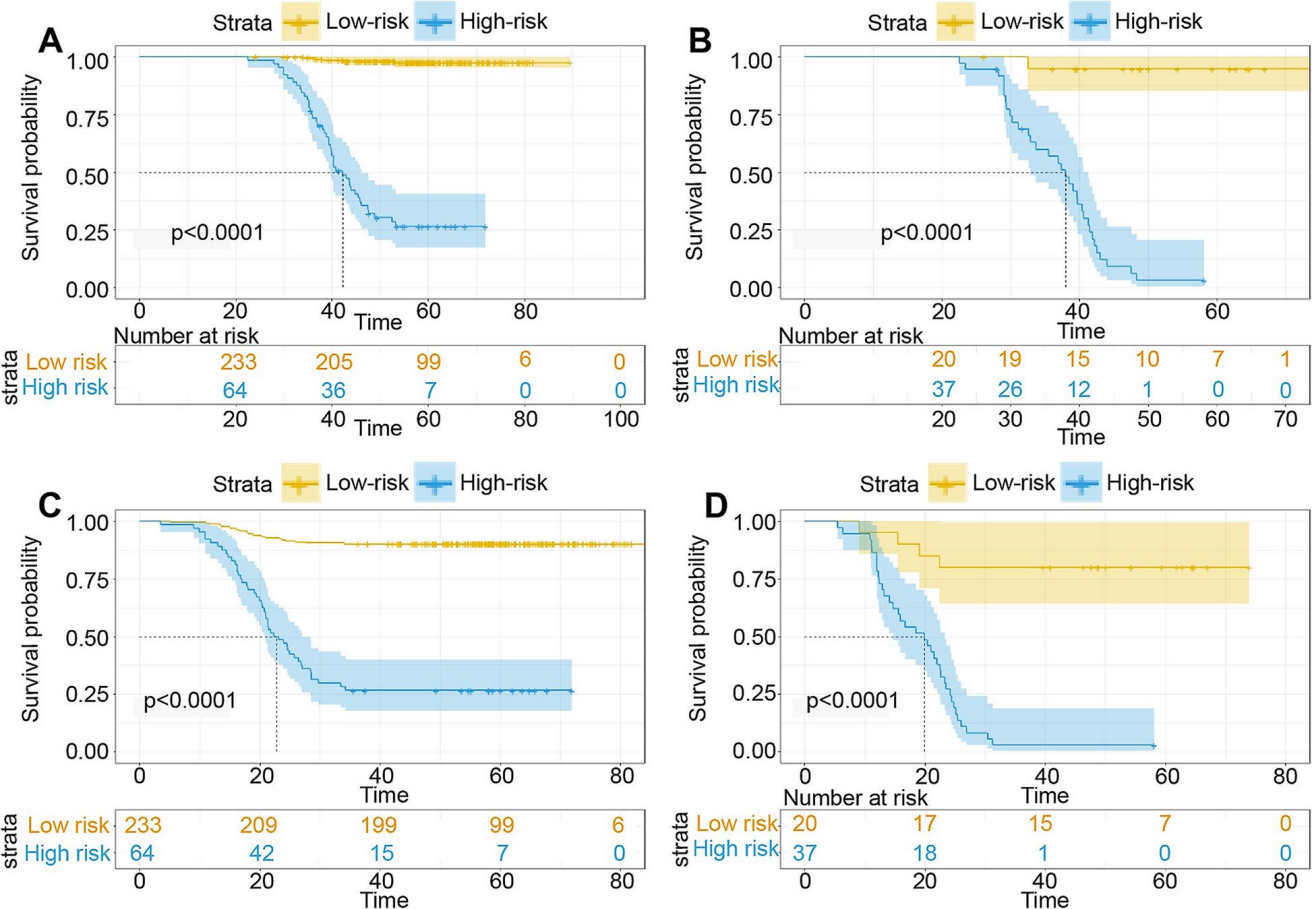
Survival curves for subgroup analysis in patients with different risk of disease-free survival (DFS) and recurrence-free survival (RFS) stratified by nomogram score. **A** Kaplan–Meier survival curves for DFS according to the risk status in lymph node metastasis (LNM)-positive patients; **B** DFS according to the risk status in LNM-negative patients. **C** RFS according to the risk status in LNM-positive patients. **D** RFS according to the risk status in LNM-negative patients

Based on the nomogram score, patients were stratified into those with low- (score ≤ 150) and high-risk (score > 150) for recurrence and mortality, respectively. We used Kaplan–Meier curves and the log-rank test to analyze disease-free survival (DFS) and recurrence-free survival (RFS) in patients with LNM or without LNM after stratification (low-risk vs. high-risk) using the nomogram. In the LNM-positive group, high-risk patients had a lower DFS and RFS (*p* < 0.001, *p* < 0.001, *p* < 0.001, and *p* < 0.001; Fig. 4A–D) than did low-risk patients.

## Discussion

LNM is a critical factor in EECs that influences their course and prognosis. Furthermore, the histological grade of an EEC is frequently considered for prognostic and therapeutic purposes. In contrast to cervical adenocarcinoma, there is currently no reliable histological grading system other than the Silva system for patients with EEC that stratifies the risk of LNM. Our study analyzed the histopathological features of EECs comprehensively to develop a nomogram that can assist clinicians in stratifying the risk of LNM. In the current study, we defined the parameters of a nomogram, which included LVI, high-grade pattern, and mitosis, for the prediction of LNM. As such, we established a novel and reliable nomogram and compared it with the traditional grading systems to show its ability to stratify the risk of LNM and to assist the surgeons in choosing the best surgical method to minimize the loss of patients during operations.

Although the traditional Silverberg and FIGO histological grading systems have been proven to have good prognostic risk stratification in many datasets [5], herein, they were unable to effectively predict LNM in patients with EEC. By integrating the original Silverberg and FIGO histological factors, we identified MELF pattern and necrosis as valuable factors for optimizing the stratification of LNM risk. Our nomogram can be stratified such that low-risk patients can avoid lymph node dissection and improve their postoperative quality of life and high-risk patients can receive appropriate postoperative adjuvant therapy.

LVI, as a traditional prognostic factor, is strongly associated with LNM; thus, it was included in the Silva grading system for cervical adenocarcinoma. A high-grade pattern is defined as solid, micropapillary, fused glands or single cells infiltrating the desmoplastic stroma. Furthermore, high-grade patterns have been shown to have prognostic significance in many cancers, including lung adenocarcinoma [6] and cervical adenocarcinoma [7]. High mitotic counts have been shown to have prognostic significance not only in cancerous tumors but also in various other tumor types. Moreover, they play an important role in differentiating benign and malignant mesenchymal tumors. However, the traditional grading system only included a high-grade pattern and high mitotic count while excluding micropapillary fused glands or single-cell patterns and LVI. The results of the present study showed that a novel nomogram that contains LVI and high-grade patterns can provide more reliable risk stratification. Furthermore, we incorporated the proportion of high-grade patterns and the number of mitotic cells as continuous variables into the nomogram; thus, this scoring system may be more objective and feasible in routine practice.

Tumor necrosis results from rapid cell proliferation and overgrowth of blood supply, leading to hypoxia in tumor cells [8]. Tumor necrosis has been demonstrated to correlate with invasiveness and adverse clinical outcomes in many cancers, including carcinomas [9], although its prognostic relevance in EECs has not yet been defined. The presence of tumor necrosis resulting in necrotic/tumor debris within the tumor gland lumen may reflect the tumor biology and provide valuable prognostic information. The association between the MELF pattern and LNM has been investigated previously [10]. Some studies have shown that patients with the MELF pattern have a higher prevalence of LNM than those without the MELF pattern [10–12]. Furthermore, the MELF pattern is related to an advanced FIGO stage and adverse histological findings [10]. These results suggest that the MELF pattern is a concomitant finding that appears in association with tumor progression. Our results agree with previously reported findings and indicate that tumor necrosis/tumors and a MELF pattern are of significant prognostic value for EECs. Although the MELF pattern and necrosis were not significant independent predictors in our multivariate analysis, their inclusion in the nomogram confirmed more accurate risk stratification.

Our study also examined the prognostic value of the tumor stroma in EC. However, this did not play a role in predicting LNM in the nomogram. Importantly, our nomogram incorporated several new histological indicators (LVI, MELF pattern, and necrosis) for ruling out high-grade nuclei. Adjuvant therapy for advanced-stage EC is also a debated topic globally. The use of radiation decreases local recurrence rates without improving distant failures; however, chemotherapy alone decreases distant failure rates without improving local control [13, 14]. Management of early-stage EC has evolved during the past two decades. The use of adjuvant treatments remains somewhat controversial, partly due to low recurrence rates after surgery alone, although upfront surgery has remained the mainstay of treatment. Pathological evaluation is an important basis for oncologists to choose postoperative adjuvant therapy, especially pathological lymph node evaluation [15]. Our model can not only effectively stratify the risk of LNM, but also predict a high risk of recurrence in patients without LNM. Patients with high-risk ECs, especially in the early stages, should receive postoperative adjuvant therapy. However, this study also has some limitations. First, the statistical power was limited because this was a retrospective single-center study. Second, owing to the retrospective study design, potential selection bias could not be excluded. Third, the sample size was small, and there was no external validation. Finally, although the study focused on identifying the most significant predictors of LNM, it is unclear whether the findings can be generalized.

## Conclusions

In summary, LVI, high-grade pattern, high mitotic counts, MELF pattern, and tumor necrosis were associated with LNM. Therefore, these features should be assessed during routine evaluation of patients with EEC, as they may provide helpful information to guide clinical therapy. Additional studies in a multi-institutional setting are needed to confirm these findings.

## Data Availability

The datasets used and analyzed during this study are available from the corresponding author upon reasonable request.

## Abbreviations

DCA: Decision curve analysis
DFS: Disease-specific survival
EEC: Endometrial endometrioid carcinoma
EC: Endometrioid carcinoma
LNM: Lymph node metastasis
LVI: Lymphovascular invasion
MELF: Microcystic, elongated, and fragmented
RFS: Recurrence-free survival
FIGO: Silverberg and International Federation of Obstetrics and Gynecology
